# Tailoring Mesoporous Silica-Coated Silver Nanoparticles and Polyurethane-Doped Films for Enhanced Antimicrobial Applications

**DOI:** 10.3390/nano14050462

**Published:** 2024-03-02

**Authors:** Silvia Nuti, Adrián Fernández-Lodeiro, Joana Galhano, Elisabete Oliveira, Maria Paula Duarte, José Luis Capelo-Martínez, Carlos Lodeiro, Javier Fernández-Lodeiro

**Affiliations:** 1 BIOSCOPE Research Group, LAQV-REQUIMTE, Chemistry Department, NOVA School of Science and Technology (FCT NOVA), Universidade NOVA de Lisboa, 2829-516 Caparica, Portugal; s.nuti@campus.fct.unl.pt (S.N.); a.lodeiro@campus.fct.unl.pt (A.F.-L.); j.galhano@campus.fct.unl.pt (J.G.); jlcm@fct.unl.pt (J.L.C.-M.); cle@fct.unl.pt (C.L.); 2 PROTEOMASS Scientific Society, Praceta Jeronimo Dias, Num. 12, 2A, Sto Antonio de Caparica, 2825-466 Costa de Caparica, Portugal; 3 MEtRICs, Chemistry Department, NOVA School of Science and Technology (FCT NOVA), Universidade NOVA de Lisboa, 2829-516 Caparica, Portugal; mpcd@fct.unl.pt

**Keywords:** silver nanoparticles, silica core shell, antimicrobial activity, polymers, bacterials, gold nanoparticles

## Abstract

The global increase in multidrug-resistant bacteria poses a challenge to public health and requires the development of new antibacterial materials. In this study, we examined the bactericidal properties of mesoporous silica-coated silver nanoparticles, varying the core sizes (ca. 28 nm and 51 nm). We also investigated gold nanoparticles (ca. 26 nm) coated with mesoporous silica as possible inert metal cores. To investigate the modification of antimicrobial activity after the surface charge change, we used silver nanoparticles with a silver core of 28 nm coated with a mesoporous shell (ca. 16 nm) and functionalized with a terminal amine group. Furthermore, we developed a facile method to create mesoporous silica-coated silver nanoparticles (Ag@mSiO_2_) doped films using polyurethane (IROGRAN^®^) as a polymer matrix via solution casting. The antibacterial effects of silver nanoparticles with different core sizes were analyzed against Gram-negative and Gram-positive bacteria relevant to the healthcare and food industry. The results demonstrated that gold nanoparticles were inert, while silver nanoparticles exhibited antibacterial effects against Gram-negative (*Escherichia coli* and *Salmonella enterica* subsp. *enterica* serovar Choleraesuis) and Gram-positive (*Bacillus cereus*) strains. In particular, the larger Ag@mSiO_2_ nanoparticles showed a minimum inhibitory concentration (MIC) and a minimum bactericidal concentration (MBC) of 18 µg/mL in the *Salmonella* strain. Furthermore, upon terminal amine functionalization, reversing the surface charge to positive values, there was a significant increase in the antibacterial activity of the NPs compared to their negative counterparts. Finally, the antimicrobial properties of the nanoparticle-doped polyurethane films revealed a substantial improvement in antibacterial efficacy. This study provides valuable information on the potential of mesoporous silica-coated silver nanoparticles and their applications in fighting multidrug-resistant bacteria, especially in the healthcare and food industries.

## 1. Introduction

Bacterial infections pose a significant challenge to public health and Gram-negative bacteria such as *E. coli* are major causes of infectious diseases worldwide [1]. These bacteria are intrinsically drug-resistant due to the presence of an impermeable outer membrane, being included in the critical category of the World Health Organization list of antibiotic-resistant priority pathogens [2,3]. *Salmonella enterica* also represents a significant challenge in animal and food safety, as it can easily spread through contaminated water and food, posing a serious health risk. The excessive use of common antibiotics has favored the spread of resistant bacteria, which develop the ability to avoid drugs designed to eliminate them. To date, many antibiotics have progressively lost their effectiveness against their targeted bacteria, which highlights the urgency needed to explore novel antibacterial agents with broad antimicrobial activity and low propensity for resistance development [4].

Silver has been appreciated for centuries as an antimicrobial material to treat infections and prevent spoilage and it is widely recognized that silver ions exhibit high toxicity to both Gram-negative and Gram-positive microorganisms. Hence, for more than 100 years, colloidal silver has been widely used as an antibacterial agent [5].

Since then, the properties of colloidal silver have been extensively investigated in this context. The last few years have seen significant advances in exploring the antibacterial activity of silver nanoparticles (AgNPs) [6], showcasing robust biocidal effects against various bacterial strains, including multi-resistant ones [7]. Moreover, commercially available products such as Acticoat^TM^ (Smith and Nephew, London, UK), Silverline^TM^ (Spiegelberg GmbH and Co. KG, Hamburg, Germany) and ON-Q Silver Soaker^TM^ (I-Flow Corporation, Lake Forest, CA, USA) [8] are also present nowadays in the commercial market, just to name a few.

AgNPs operate through diverse mechanisms encompassing the Ag^+^ ions, catalysis of the production of reactive oxygen species (ROS), and disruption of the cellular barrier of the bacteria by accumulating on their surface, leading to oxidative stress, damage to bacterial membranes, and interference with cellular processes, ultimately resulting in bacterial growth inhibition or cell death [9]. Furthermore, the size of AgNPs shows a size-dependent antimicrobial nature which can be associated with the higher oxidation rate and increased cell permeability as the size is decreased [10]. Others physicochemical properties of AgNPs have been pointed as key factors in their antibacterial activity, such as surface charge or the molecular entities that stabilized them [11,12,13].

Beyond their role in biomedicine, AgNPs also find widespread application in textiles, cosmetics, and the food industry [14]. However, challenges persist, particularly in terms of colloidal stability in biological mediums and susceptibility to oxidation, limiting their consistent application [15]. Cell permeability, especially with smaller free nanoparticles, demands careful consideration in product development. Addressing these issues, the introduction of a coating becomes essential to prevent aggregation and excessive oxidation, thereby ensuring sustained and more prolonged antibacterial performance. Such coating would enable the regulation of the release of ionic silver (Ag^+^) into a solution, followed by organic functionalization and enhanced biocompatibility and stability in biological media. This approach could prove to be a potent strategy to ensure a safer use of AgNPs as antibacterial agents. Various coatings for AgNPs have been investigated, including polymers or metal oxides [16]. Among these, mesoporous silica presents several advantages. Firstly, it enhances the stability of AgNPs, preventing uncontrolled oxidation of the silver core. Additionally, it improves biocompatibility and facilitates applications in drug release by leveraging its porous mesostructure as a reservoir for organic molecules. Lastly, functionalizing the external surface with silanes allows for the modification of surface properties, making them compatible with a broad range of applications [17,18,19,20]. Importantly, by manipulating the final size through silica coating, skin penetration and cellular uptake can be avoided [21], as they constitute important issues in integrating nanoparticles into future products with antimicrobial properties for daily use. While the potential of mesoporous silica-coated silver (Ag@mSiO_2_) NPs is recognized, the scarcity of well-defined synthesis methodologies with high yields [22,23,24] has hindered comprehensive exploration of their antimicrobial activity. The limited body of literature on this subject highlights the need for further research to unlock the full potential of these materials.

Recently, we have devised a synthetic method to precisely coat silver nanoparticles with mesoporous silica in high yield, offering the flexibility to adjust both the core size and the thickness of the silica coating [12]. While achieving meticulous control over the produced colloids, challenges arise in their practical application due to limitations in the quantity of nanomaterial that can be obtained. Therefore, research focused on enhancing the production yield while upholding high quality is imperative. This research not only seeks to deepen our understanding of the antibacterial properties of mesoporous silica-coated silver nanoparticles, but also stimulate innovative ideas for their seamless integration into advanced materials.

Regarding the antibacterial properties of Ag@mSiO_2_, to the best of our knowledge, there are no studies that have investigated the antibacterial effects of Ag@mSiO_2_ against *Salmonella enterica* subsp. *enterica* serovar Choleraesuis. However, Neethu et al. [25] conducted an antibacterial study using uncoated AgNPs against *Salmonella enterica* serovar Typhimurium, achieving a minimum bactericidal concentration (MBC) value of 16 µg/mL. It is worth noting that all the studies conducted so far have primarily focused on uncoated AgNPs, and it is expected that the addition of an extra layer of mesoporous silica will slow down the release of silver ions. This may improve the efficacy of the AgNPs as antibacterial agents, as well as reducing the potential for bacterial resistance development.

With the aim of harnessing the antimicrobial properties of metal nanoparticles, they have previously been widely employed as dopants in various polymeric matrices. This approach is undertaken to develop polymeric surfaces endowed with antimicrobial properties. Among the diverse polymer matrices, thermoplastic polyurethanes (TPU) constitute a versatile class of polymeric materials widely utilized across various applications due to their exceptional physical properties. These properties include resistance to chemicals, radiation, weathering, cut/tear, abrasion, and scratches, as well as elasticity, transparency, and impact strength. Their applications span diverse sectors, including automotive, sensors, food packaging, and medical devices, among others [26,27].

With an antibacterial approach, TPU matrices doped with Cu or Ag nanoparticles have demonstrated to have better antibacterial properties compared to the undoped TPU, showcasing the potential for enhancing the properties of these polymers through the addition of metallic nanomaterials [28]. Furthermore, combinations of TPU and polylactic acid doped with Ag NPS have been reported and tested for preserving fruits while exhibiting antibacterial properties against food-related bacteria, thereby contributing to the advancement of antibacterial food packaging [29,30,31].

This study delves into the antimicrobial properties of silver nanoparticles of two different sizes coated with mesoporous silica against common pathogens found in health and food-related settings worldwide. Additionally, we explore the effects of modifying the surface charge of NPs and their application in producing polyurethane films doped with Ag@mSiO_2_ NPs.

To manufacture the colloids for this study, we explored the effect of scaling-up our previous synthetic process by up to four times to achieve a large quantity of well-defined core–shell nanoparticles in high yield [12]. Furthermore, we synthesized mesoporous silica-coated gold NPs to use them as a control and to discern the antimicrobial effects produced by the silver cores compared with a neutral metallic core. Functionalization with amine propyl triethoxy silane (APTES) as well as its integration into polyurethane films have been investigated. Subsequently, the antibacterial efficacy against *Escherichia coli*, *Salmonella enterica* subsp. *enterica* serovar Choleraesuis and *Bacillus cereus* of the produced materials were assessed.

## 2. Materials and Methods

### 2.1. Materials

Silver(I) nitrate (AgNO_3_) (99.9%), trisodium citrate dihydrate (C_6_H_5_Na_3_O_7_·2H_2_O, ≥99.5%), Adenosine monophosphate (99%), Gold(III) chloride trihydrate (HAuCl_4_·3H_2_O, 99.9%), sodium hydroxide (NaOH, BioXtra ≥ 98%, pellets, anhydrous), absolute ethanol (CH_3_CH_2_OH, ≥99.8%) and absolute methanol (CH_3_OH ≥ 99.9%) were obtained from Merck. Tannic acid (98%), Tetraethoxysilane (C_8_H_20_O_4_Si, 99+%), (3-Aminopropyl) triethoxysilane (C_9_H_23_NO_3_Si, 97%) were obtained from Alfa Aesar. Hexadecyltrimethylammonium Chloride (C_19_H_42_ClN, 95%) was obtained from TCI Chemicals. IROGRAN^®^ A92P4637 was provided by Huntsman GmbH (Osnabruck, Germany). All reagents were used as received without further purification. Ultrapure water (type I) was used for the preparation of all the water-based solutions. The glassware was cleaned with aqua regia and rinsed with ultrapure water prior to the experiments.

### 2.2. Instrumentation

The extinction spectra were recorded using a JASCO 770 UV−Vis-NIR spectrophotometer provided by the PROTEOMASS-BIOSCOPE facility (Caparica, Portugal). All spectra were recorded using a HELMA 1 cm light path quartz cell. z-potential analyses were carried out in a Malvern ZS instrument at 22 °C provided by the PROTEOMASS-BIOSCOPE facility (Caparica, Portugal). Low-magnification transmission electron microscopy (TEM) images were obtained using a JEOL JEM 1010 TEM microscope (JEOL, Tokyo, Japan), working at 100 kV. High Resolution Transmission Electron Microscopy (HRTEM), Scanning Transmission Electron Microscopy (STEM), Energy Dispersive X-ray Spectrometry (EDX), Scanning Electron Microscope (SEM) and X-ray photoelectron spectroscopy (XPS) analyses were provided by the INL facility in Braga, Portugal. The SEM images of the top-view of the polymer were obtained with the polymers without gold coating, while the images of the cross section were acquired after the deposition of Au for 40 s.

### 2.3. Methods

#### 2.3.1. Synthesis of AgNPs

AgNPs with a mean size of 28.1 ± 1.9 nm were synthesized with the following route: 200 mL of an aqueous solution containing tannic acid (TA) 0.1 mM and sodium citrate (SC) 5 mM were placed in a double neck round-bottom flask adapted with a condenser. The solution was heated under vigorous stirring and, after boiling for 15 min, 2 mL of AgNO_3_ (25 mM) was quickly injected, causing an immediate change to yellow color. The reaction was considered complete after boiling for 45 min; then, the solution was cooled under stirring at room temperature.

A total of 100 mL of this colloidal solution was purified through centrifugation (8500 rpm × 30 min), to remove the TA and SC excess, and redispersed in 90 mL of SC 2.2 mM for further functionalization.

To synthesize AgNPs with a mean size of ca. 51.2 ± 4.1 nm, 100 mL of the as-obtained AgNPs (28 nm) were used as seeds and 3 growth steps were performed at a temperature of 90 °C, as previously reported by N. Bastus et al. [32] After the growth, the bigger AgNPs were purified through centrifugation (5500 rpm × 30 min), to remove the TA and SC excess, and redispersed in 90 mL of SC 2.2 mM for further functionalization.

#### 2.3.2. Synthesis of AuNPs

AuNPs with a mean size of 25.3 ± 2.3 nm were obtained with a direct synthesis based on the report by I. Ojea-Jiménez et al., with some modifications [33]. Briefly, 149 mL of an aqueous solution of HAuCl_4_·3H_2_O 0.215 mM was placed in a two-necks round-bottom flask adapted with a condenser, then heated and left boiling for 15 min. Then, 1 mL of SC 0.3 M was added to reach a final reaction volume of 150 mL. The reaction was left boiling until an intense red-wine color was obtained (ca. 30 min); after cooling down while stirring at room temperature, 100 mL of the colloidal solution was purified through centrifugation (8000 rpm × 30 min) and redispersed in 90 mL of SC 2.2 mM for further functionalization.

#### 2.3.3. Functionalization of AgNPs and AuNPs with AMP

AgNPs and AuNPs were synthesized and purified as described above. The solution was transferred to a round-bottom flask and 10 mL of an aqueous solution of AMP (50 mM) was dripped into the solution under vigorous magnetic stirring. The solution was ultrasonicated for 3 min and then stirred for 48 h at room temperature. Finally, AgNPs and AuNPs were centrifuged and redispersed in NaOH 2 mM to reach a concentration of ~5.8 × 10^11^ NPs/mL for silver colloids and ~6.1 × 10^11^ NPs/mL for gold colloids. The so-obtained solutions were stored at 4 °C for further use. The nanoparticle concentrations (NPs/mL) were determined considering a quantitative metal reduction synthesis. To calculate the total volume of silver (Ag) or gold (Au) used, we utilized the density of face-centered cubic (fcc) structures and the atomic mass for silver (10.5 g/cm^3^ and 107.9 g/mol, respectively) and gold (19.3 g/cm^3^ and 197 g/mol). Mean particle sizes, extrapolated from transmission electron microscope (TEM) images, were considered. The volume of a single nanoparticle was computed using V_sphere_ = (4/3)π(r)^3^, where ‘r’ represents the radius. The total number of nanoparticles was estimated by dividing the volume of metal used by the volume of a single nanoparticle. The final nanoparticle concentration was then determined based on the final volume of solution using for nanomaterial resuspension [34].

#### 2.3.4. Mesoporous Silica Coating of AgNPs or AuNPs

A total of 97.28 mL of water and 1920 µL of CTAC 50 mM were placed in a round-bottom flask. Then, 20 mL of NPs in NaOH 2 mM (in concentration of ~5.8 × 10^11^ NPs/mL for Ag and 6.1 × 10^11^ NPs/mL for Au) was dropped in the reaction under vigorous magnetic stirring. After 5 min, 800 µL of NaOH 0.1 M was injected, and the solution was moderately stirred for 24 h. Afterwards, 4 mL of EtOH containing TEOS was sequentially dripped (800 µL each 10 min) under stirring (small Ag or Au NPs were coated using 80 µL of TEOS, while for bigger AgNPs 100 µL was used). The bottom flask was capped, and moderate stirring was maintained for 24 h at room temperature. Finally, the Ag@mSiO_2_ and Au@mSiO_2_ NPs were purified with 3 centrifugation cycles (rpm range 9000–6500 × 30 min) in EtOH for microscopy analysis. The colloid solutions were stored in EtOH at 4 °C for further use. The reaction yield was obtained by counting a minimum of 200 NPs per sample to determine the percentage of well-defined Ag@mSiO_2_ or Au@mSiO_2_ NPs. To substantially eliminate the ammonium template, the NPs underwent further purification through three additional rounds of centrifugation at 9500 rpm for 30 min in MeOH, followed by two additional cycles in water. Ultrasonication for ca. 3 min was applied to the colloid solutions between each centrifugation round.

#### 2.3.5. Amine Derivatization of Ag(28)@mSiO_2_-OH to Ag(28)@mSiO_2_-NH_2_

First, Ag(28)@mSiO_2_ core–shell nanoparticles were synthesized following the previously outlined procedure. The obtained nanoparticles underwent purification through two centrifugation cycles (at 9500 rpm for 30 min) in EtOH. After the second cycle, the pellets were resuspended in 60 mL of EtOH with a [H_2_O] concentration of 5.55 M (composed of 54 mL of absolute EtOH and 6 mL of H_2_O).

Subsequently, 1 mL of EtOH containing 1.3 mL of APTES was added, and the reaction proceeded for 6 h. Ultrasonication was applied for 3 min at 2 h intervals during the reaction time. Finally, the resulting nanoparticles were subjected to further purification through repeated cycles as explained above.

#### 2.3.6. Ag(28)@mSiO_2_-NH_2_ Doped Polymer Synthesis

The polymer was synthesized using Ag(28)@mSiO_2_-NH_2_, along with IROGRAN^®^ as the polymer matrix. The process involved the obtainment of Ag(28)@mSiO_2_-NH_2_, followed by purification in EtOH and MeOH as explained previously. Subsequently, the NPs were transferred to tetrahydrofuran (THF). The concentrated THF solution was then diluted to 100 µg/mL of silver using ICP analysis.

The nanoparticles solutions in THF were utilized to create the polymer film. Briefly, 5 mL of THF containing 60 mg/mL of IROGRAN^®^ was mixed with varying volumes of the concentrated NP solutions in THF (2 or 4 mL). After mixing the polymer solution with different volumes of NP solutions, the samples were placed in a capped vial and manually shaken for homogeneity. Finally, the samples underwent 3 min of ultrasonication to remove bubbles and were deposited in a crystal Petri dish measuring 8 cm in diameter. The solution was allowed to evaporate, resulting in the formation of a thin film.

#### 2.3.7. Antimicrobial Assays

The antibacterial activity of the nanoparticles was evaluated against *Escherichia coli* (*E. coli* 2, ATCC^®^ 25922^TM^), *Salmonella enterica* subsp. *enterica* serovar Choleraesuis (*Salmonella* Choleraesuis, ATCC^®^ 10708^TM^), and *Bacillus cereus* (*B. cereus*, ATCC^®^ 11778). Bacteria stocks were kept frozen at −70 °C in broth containing glycerol (15% *v*/*v*). Initial bacterial suspensions of all strains were inoculated in Tryptone Soy Agar (TSA, Biokar, Allonne, France) and incubated overnight at 35 ± 2 °C (*E. coli* and *Salmonella* Choleraesuis) or 30 ± 2 °C (*B. cereus*). Isolated colonies were transferred to saline medium (NaCl, 0.85% *m*/*v*) and the turbidity of the suspension was adjusted to 0.5 on the McFarland scale (approx. 1.5 × 10^8^ CFU/mL) (DEN-1B McFarland Densitometer, Grant-bio, Cambridge, UK).

The antibacterial activity of the nanoparticles, Ag(28)@mSiO_2_, Ag(51)@mSiO_2_, Au@mSiO_2_ and Ag(28)@mSiO_2_-NH_2,_ was assessed using the broth microdilution method in 96-well microplates following metal concentration into the nanoparticles ranging from 0.1 to 78 µg/mL. Each well was inoculated with the above prepared bacterial suspension (10^6^ CFU/mL in each well). The microplates were incubated, in the dark, at 35 ± 2 °C (*E. coli* and *Salmonella* Choleraesuis) or 30 ± 2 °C (*B. cereus*) for 24 h. Upon the appointed time, an aliquot of each well was spread onto TSA *Petri* dishes and incubated in the dark, for an additional 24 h at 35 ± 2 °C (*E. coli* and *Salmonella* Choleraesuis) or 30 ± 2 °C (*B. cereus*).

The minimum inhibitory concentration (MIC) was assessed using optical density (OD, 600 nm) quantification (UV–Vis CLARIOstar spectrophotometer, BMG Labtech, Ortenberg, Germany). A MIC was considered for a reduction in turbidity/viability of approx. 50% compared to the control. The minimum bactericidal concentration (MBC) was the lowest nanoparticle concentration that inhibited bacterial growth in TSA plates inoculated with the aliquots taken from the microplate wells.

To assess the antimicrobial activity of Ag(28)@mSiO_2_-NH_2_ doped polymers, microbial suspensions, prepared as previously described, were spread on the surface of Mueller–Hinton agar plates (Biokar, Allonne, France). Then, squares of non-doped and nanoparticle-doped-polyurethane films (ca. 1 cm^2^ in area) were applied and the plates were incubated in the dark, for 24 h at 30 ± 2 °C (*Bacillus cereus*) or 35 ± 2 °C (remaining microorganisms). Antimicrobial activity was evaluated by observing the bacterial growth in the polymer–agar interface.

Asepsis was achieved in a VBH Laminar Flow Hood (Steril). Incubations were carried out in a Mermmet Incubator B50 (Vikat Ekinox, Wormhout, France).

## 3. Results and Discussion

### 3.1. Synthesis and Characterization of Mesoporous Silica Coating Ag and Au NPs

To synthetize the metallic NPs, reproducible high yield syntheses of AgNPs and AuNPs via metal reduction with tannic acid and/or sodium citrate were applied with minor modifications [12,32,33] (see Section 2). We have manufactured AgNPs with 28.1 and 51.2 nm showing a LSPR band centered in ca. 406 nm and 415 nm, respectively (see Figure S1). In the case of gold, NPs with a LSPR band in ca. 520 nm and a mean size of 25.3 nm were obtained (Figure S2).

We have recently reported the benefits of the functionalization of AgNPs with adenosine monophosphate (AMP) for their subsequent silica coating [12]. In the current study, all the colloids (Ag and Au) were functionalized with AMP for their subsequent use as seeds in the growth of mesoporous silica. Our intention was to ensure uniform conditions of functionalization across all samples, thereby guaranteeing that any observed differences in the antimicrobial effect would arise from the metallic cores themselves, rather than from variations in the organic entities used for functionalizing the nanoparticles. The NPs were functionalized with AMP through incubation, and after purification, the colloids were resuspended in NaOH 2 mM at a concentration of ~5.8 × 10^11^ NPs/mL for silver colloids and 6.1 × 10^11^ NPs/mL for gold colloids (see Section 2). These colloids were then utilized as seeds in the subsequent coating experiments.

To obtain a large quantity of NPs, we expanded the coating reaction four-fold compared to our previous report [12], performing the coating reaction in a final volume of 120 mL in all cases. CTAC was used as the template, and the pH was adjusted to 10.5–11 using NaOH. We previously observed that the thickness of mesoporous silica can be manipulated by changing the [TEOS] or by varying the [CTAC] while keeping [TEOS] constant. Here, to maintain the same synthetic conditions in all cases, we have selected a [CTAC] of 0.8 mM regardless of the size or the metal core used as seed, and we have varied the [TEOS] to produce a relatively comparable silica thickness in all cases.

**Figure 1:**
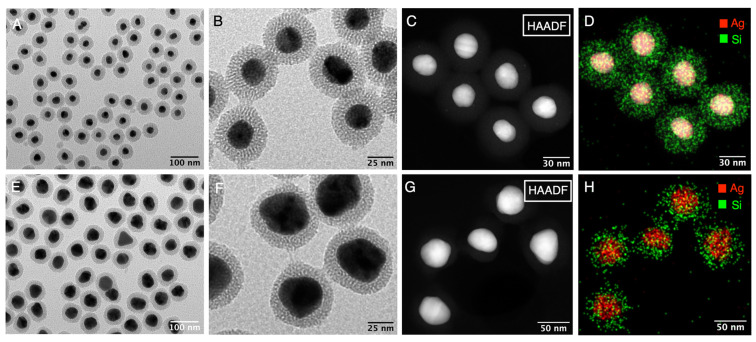
(**A**,**B**) Low-magnification TEM images of Ag(28)@mSiO_2_. (**C**) High-magnification HAADF-STEM image and (**D**) Ag and Si distribution EDX maps of Ag(28)@mSiO_2_. (**E**,**F**) Low-magnification TEM images of Ag(51)@mSiO_2_. (**C**,**G**) High-magnification HAADF-STEM image and (**D**,**H**) Ag and Si distribution EDX maps of Ag(28)@mSiO_2_ and Ag(51)@mSiO_2_ respectively.

The TEM images showed that the synthesis of Ag@mSiO_2_ was successful in manufacturing a large quantity of nanoparticles in high yield (~95%), comparable with our previous report (Figure 1A,B,E,F). In the selected condition, we obtained a mean SiO_2_ thickness of ca. 16.1 and 16.5 nm for Ag(28)@mSiO_2_ and Ag(51)@mSiO_2_, respectively. The composition of the core–shell structure, with the silver in the core and silica in the shell, were confirmed through the EDX-STEM analysis (Figure 1C,D,G,H).

**Figure 2:**
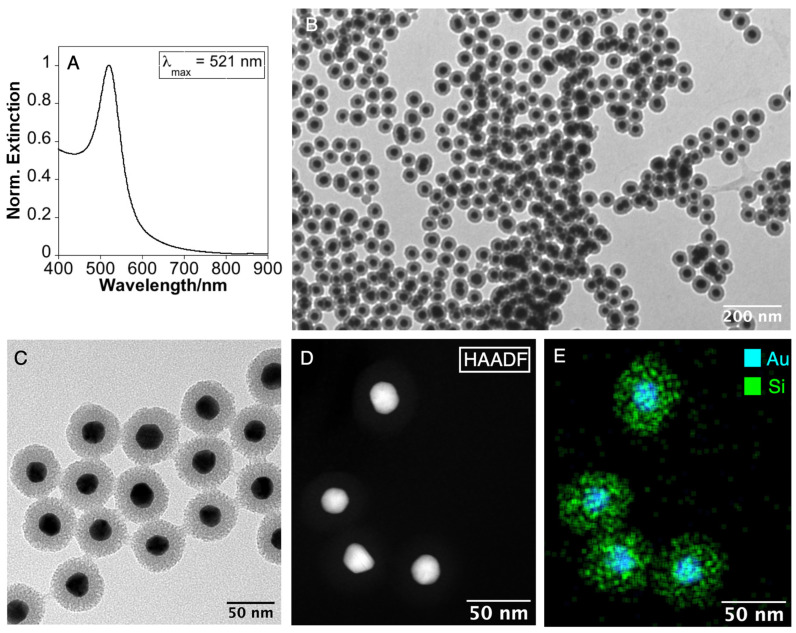
(**A**) UV-Vis extinction spectrum of Au NPs. (**B**,**C**) Low-magnification TEM images of Au@mSiO_2_. (**D**) High-magnification HAADF-STEM image and (**E**) Au and Si distribution EDX maps of Au@mSiO_2_ NPs.

When the coating reaction was explored using AuNPs as seeds, a homogeneous mesoporous silica layer with a mean thickness of ca. 16.4 nm was obtained. Our results reveal the flexibility of these coating conditions to produce a large quantity of well-defined core–shell NPs in high yield, using AMP functionalized gold or silver NPs as seeds (Figure 2).

To prepare the colloids for the exploration of their antimicrobial properties, the NPs were purified via subsequent centrifugation steps in EtOH, MeOH and H_2_O. UV-Vis spectroscopy and ζ-potential were used to confirm the substantial removal of CTAC. As an example, Figure S2 shows the UV-Vis spectra of Ag(51)@mSiO_2_ and Au@mSiO_2_ taken at different stages of the purification cycle with MeOH, upon which a blue shift of the LSPR band was observed in all cases. The blue shift was accompanied by a decrease to more negative values of the ζ-potential, reaching a value of ca. −38 mV after purification in MeOH and water (Figure S3). These observations are consistent with what has been observed in other silver and gold nanoparticles coated with silica, for which this blue shift has been associated with a substantial removal of CTA^+^ molecules that can remain between the metal surface and the silica layer and/or in the pores of this layer [12,35,36].

**Figure 3:**
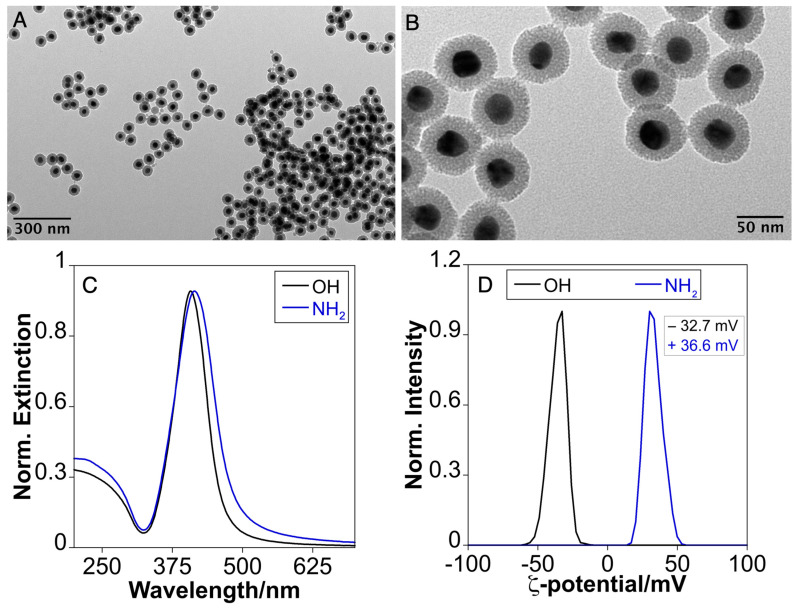
(**A**,**B**) Low-magnification TEM images of Ag(28)@mSiO_2_-NH_2_, (**C**) UV-Vis extinction spectrum and (**D**) ζ-potential of purified Ag(28)@mSiO_2_-OH (black) and Ag(28)@mSiO_2_-NH_2_ (blue).

To investigate the impact on the surface charge on antibacterial activity, we derivatized the terminal -OH group of Ag(28)@mSiO_2_ by converting it into -NH_2_ through a silane-coupling reaction using APTES (see Section 2). After the reaction, the purified Ag(28)@mSiO_2_-NH_2_ did not show a relevant increase in silica thickness (Figure 3A,B). The optical properties showed a redshift with λ_max_ of 412 nm. Furthermore, the purified nanoparticles underwent a reversal of their surface charge, reaching a ζ-potential of +36.6 mV. This shift indicates a functionalization with terminal amine groups, which become ionized in water as -NH_3_^+^.

Unlike unmodified water-soluble AgNPs, the encapsulation of these nanoparticles with mesoporous silica is anticipated to enhance solubility in a diverse array of solvents [37], enabling their integration into hydrophobic polymeric matrices via solution casting methodologies [38]. This approach holds promise for developing antibacterial films and surfaces utilizing readily available polymeric matrices. Considering the diverse applications of thermoplastic polyurethane (TPU) in fields such as medicine [39,40] and the food industry [41,42], among others, prior research has primarily focused on the doping or surface deposition of silver nanoparticles in various polyurethane matrices. In general, the manufacturing strategies for incorporating AgNPs into TPU polymeric matrices involve post-doping. This includes the specific functionalization of the AgNPs or custom construction of TPU matrices blending with other polymers or molecules possessing functional groups that enhance interaction with the AgNPs. Additionally, methods such as the direct reduction of silver salts within the polymer matrix are employed, among other approaches [43,44,45,46].

As proof of concept, we investigated the incorporation of Ag@mSiO_2_-NH_2_ NPs into TPU films utilizing IROGRAN^®^, a polyether-based thermoplastic polyurethane soluble in THF, to create polymer films doped with two amounts of nanoparticles. The NPs were synthesized and amine derivatized, purified, and finally resuspended in a concentrated THF solution. The silver concentration of this solution was adjusted to 100 μg/mL of silver using inductively-coupled plasma mass spectrometry (ICP-MS). Different volumes of this THF solution were mixed with a 5 mL THF solution of the polymer (60 mg/mL). The resulting yellow solutions were then poured into a crystal Petri dish and left to evaporate (see Section 2). After THF evaporation, homogeneous thin films were obtained.

We manufactured doped films with an area of 50.24 cm^2^ using 2 and 4 mL of concentrated Ag(28)@mSiO_2_ THF solution, providing a silver concentration of 4.0 and 8.0 μg/cm^2^, respectively.

**Figure 4:**
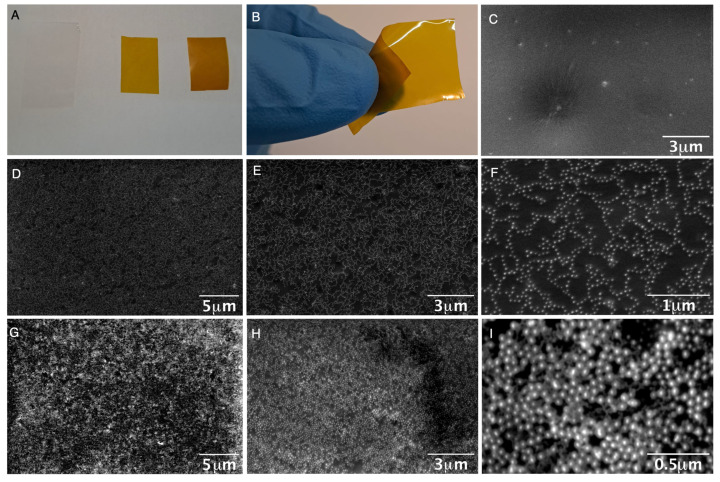
(**A**) Undoped film (transparent film) and doped film with different quantity of Ag(28)@mSiO_2_-NH_2_ nanoparticles (yellow films). (**B**) Flexibility of the doped film. (**C**,**D**) Scanning Electron Microscope (SEM) top-view images of the undoped film (**C**) and doped film with 4 μg/cm^2^ (**D**–**F**) and 8 μg/cm^2^ (**G**–**I**) at different magnifications (10K×: (**D**,**G**); 20K×: (**C**,**E**,**H**); 80K×: (**F**) and 160K×: (**I**)).

The doped films exhibited a homogeneous deep yellow color and flexibility apparently similar to their undoped counterpart (Figure 4A,B). The detailed top-view SEM images were obtained to provide a close-up perspective of the surfaces of the fabricated polymers. The undoped polymer film exhibited a homogeneous surface (Figure 4C). Notably, the scanning electron microscopy (SEM) images of the doped films revealed the presence of abundant and well-dispersed core–shell NPs within the polymer film, forming an interconnected network reminiscent of chains across the entire polymer surface (Figure 4D). The SEM images at a higher magnification clearly showed the silver cores on the polymer surface, while the silica shell remained less visible (Figure 4E,F). This suggests complete integration into the polymer matrix without exposing the NPs to the surface. In contrast, films produced with an increased nanoparticle concentration exhibited specific regions where exposure of the core–shell nanoparticles occurred, as evident in SEM images at high-magnification, clearly showing the core–shell structure of the NPs (Figure 4G–I).

**Figure 5:**
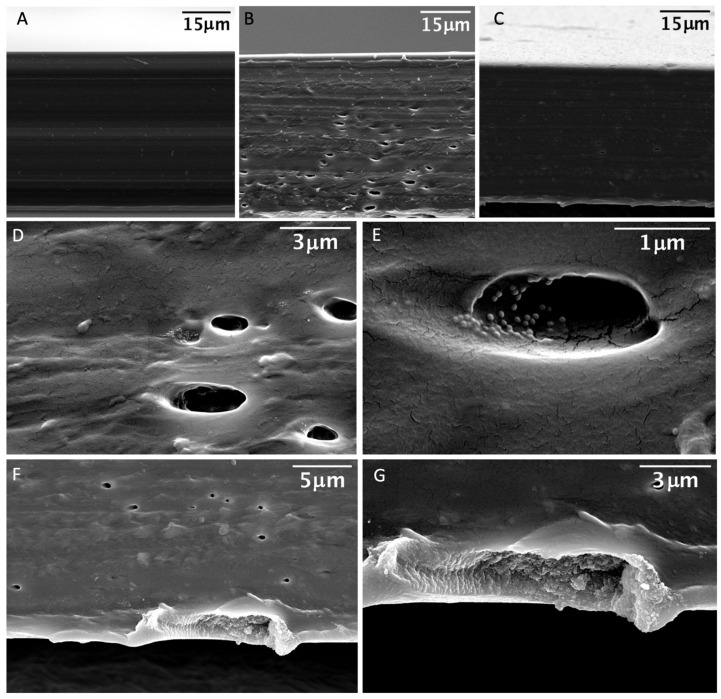
(**A**) SEM images of the cross-section view of the undoped polymer and doped film with 4 μg/cm^2^ (**B**) and 8 μg/cm^2^ (**C**). SEM images at different magnification of different areas of the cross section where the nanoparticles are exposed towards the surface for the film doped with 4 μg/cm^2^ (**D**,**E**) and 8 μg/cm^2^ (**F**,**G**).

To investigate the internal structure of the fabricated polymers, SEM images were acquired from cross sections of the films, obtained by carefully cutting the polymer. Surprisingly, while the undoped polymer exhibited a uniform internal structure (Figure 5A), the doped polymers revealed the presence of internal cavities (Figure 5B,C). A more detailed examination through higher resolution SEM images showed the core–shell NPs both in the exposed areas after cutting and on the walls of the internal cavities. In the case of the polymer doped with a higher concentration of NPs, its internal structure displayed a remarkably abundant concentration of NPs, as demonstrated by the SEM images obtained in the breakdown areas close to the cross section. Our findings indicate the comprehensive integration of the core–shell nanostructure within the doped films, resulting in effective impregnation of the films both on the surface and internally. This homogeneous distribution holds promise for applications requiring antimicrobial properties.

### 3.2. Antimicrobial Assays

**Figure 6:**
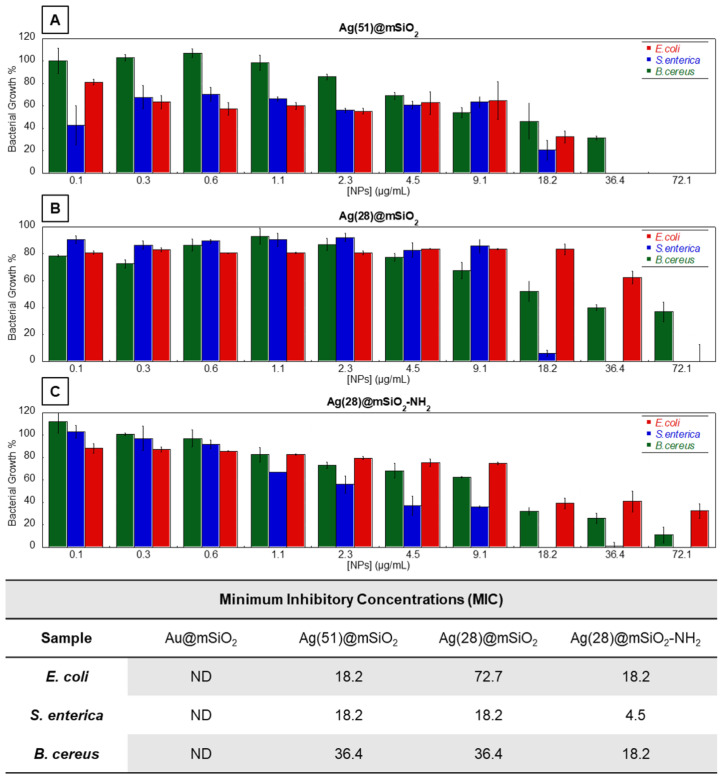
(**A**–**C**) Bacterial growth profiles, against Gram-negative bacteria (Red) *Escherichia coli* (*E. coli*, ATCC^®^ 25922^TM^), (Blue) *Salmonella enterica* subsp. *enterica* serovar Choleraesuis (ATCC^®^ 10708^TM^) and Gram-positive bacteria (Green) *Bacillus cereus* (ATCC^®^ 11778^TM^). Concentrations are relative to the metal concentration in the nanoparticles previously determined via ICP. Obtained MICs for all nanoparticles against the tested Gram-negative and Gram-positive bacteria. In the case of *S. enterica* the obtained MICs correspond to the MBC values, as well. NPs: Au@mSiO_2_; Ag(51)@mSiO_2_; Ag(28)@mSiO_2_; Ag(28)@mSiO_2_-NH_2_.

A primary study was performed to evaluate the antimicrobial activity of Au@mSiO_2_, Ag(28)@mSiO_2_, Ag(28)@mSiO_2_-NH_2_ and Ag(51)@mSiO_2_ NPs against bacterial strains that are commonly reported as medical and food contaminants. These strains included *Escherichia coli* (*E. coli*, ATCC^®^ 25922^TM^), *Salmonella enterica* subsp. *enterica* serovar Choleraesuis (ATCC^®^ 10708^TM^) and *Bacillus cereus* (ATCC^®^ 11778^TM^). Two different indicators were used to evaluate the antibacterial activity of the nanoparticles, the Minimum Inhibitory Concentration (MIC) and Minimum Bactericidal Concentration (MBC). The MIC_50_ is herein described as the concentration of nanoparticles that can derive an inhibition of growth equal to or greater than 50%. These values are determined through the plots obtained and represented in Figure 6. As for the MBC values indicated, these values are representative of the concentration of nanoparticles that can produce a bactericidal effect in each strain and are determined via the collection and plating of an aliquot of each individual tested condition. These values were determined through a viability test, in which a drop of each individual condition was replated on an agar plate without the presence of the nanoparticles. If no bacterial growth was observed following an appropriate incubation period, that condition would be determined as the MBC.

A broth microdilution methodology was used to determine the minimum inhibitory concentration (MIC_50_) values of the NPs for each bacterial strain.

Before the antimicrobial studies, the NPs underwent several purification cycles in methanol and finally water, and their concentration was evaluated based on the silver concentration, determined using ICP-MS.

Bacterial strains were incubated overnight with a gradient concentration of each sample ranging from 0.1 to 72.1 µg/mL. Figure 6 summarizes the obtained results, such as bacterial growth profiles and the MIC values for the tested AgNPs. As expected, Au@mSiO_2_ NPs did not exhibit any antibacterial effects against the selected strains, due to the inert bioactive profile of the gold core as we have reported recently [47].

Overall, the tested AgNPs exhibited a strong antibacterial activity against both Gram-positive and Gram-negative bacteria, with a broad-spectrum inhibitory activity. Interestingly, the size of the silver core appears to have an important effect on the antibacterial activity that the particles produce, when comparing the Ag(51)@mSiO_2_ and Ag(28)@mSiO_2_ NPs. Although a comparable silica coating was obtained for both silver core sizes, and a more pronounced oxidation rate would be expected with the smaller nanoparticles, the larger ones, Ag(51)@mSiO_2_, showed an improved inhibitory activity when in comparison to the smaller, Ag(28)@mSiO_2_, against Gram-negative bacteria. In relation to *E. coli*, the determined MIC values were 18.2 µg/mL for Ag(51)@mSiO_2_ and 72.7 µg/mL for Ag(28)@mSiO_2_. For *Salmonella* Choleraesuis, the MIC values for both nanoparticles were 18.2 μg/mL. At this same concentration we observed the existence of a bactericidal effect, in not only the MIC but also the MBC. These values indicate the occurrence of some selectivity mechanisms which are not present for Gram-positive bacteria, as the MIC values obtained for both large and small nanoparticles were similar for *B. cereus* (MIC = 36.4 µg/mL).

Despite variations in size, the silver nuclei employed in this study display a consistent polycrystalline structure, as illustrated in Figure S4. The diverse antimicrobial effects observed among the investigated nanoparticles do not appear to correlate with the distinct crystalline structures present in the samples explored. Furthermore, to delve into the physicochemical distinctions among the investigated nanoparticles and their correlation with the observed variations in antimicrobial efficacy, we employed X-ray photoelectron spectroscopy (XPS). The survey spectrum of samples confirms the presence of silver, silicon and oxygen through the respective binding energies (see Figure S5). The enlarged spectrum in the Ag region exhibits the characteristic 3D doublet structure with the typical binding energy of 368.5 and 368.4 eV for Ag(28)@mSiO_2_ and Ag(51)@mSiO_2_ NPs, respectively, and a doublet splitting at +6 eV in both cases (see Figure S6). It can be pointed out that the deconvoluted spectrum of the 3D doublet consists of a single spectrum, which can be assigned to the presence of Ag(0) (see Figure S6B,E) [48]. Confirmation of the absence of oxidized silver is derived from oxygen spectra. Figure S6C,F illustrates the central core-level emission of O 1s for both samples. Both exhibit comparable O 1s spectra, with a distinct peak only at 533.1 eV and 533.0 eV for Ag(28)@mSiO_2_ and Ag(51)@mSiO_2_, respectively, indicative of SiO_2_ [49].

Considering the total size of Ag(28)@mSiO_2_ and Ag(51)@mSiO_2_ NPs, ca. 60 and 83 nm, respectively, their antimicrobial properties associated with cell penetration effects can be excluded in both cases [10,21]. The homogeneous coating with mesoporous silica offers similar exposed organic groups but with a greater surface area per NP in the case of Ag(51)@mSiO_2_ NPs. Furthermore, larger nanoparticles, when in solution, tend to fuse and agglomerate more than their smaller counterparts [50], which may contribute to the increase in the concentration of NPs deposited on the surface of the bacteria, enhancing their antibacterial effect.

Although the obtained results point to the existence of a size dependence in the antibacterial effect, the surface charge of the particles also plays a key role in the bioactive effects of silica-coated silver nanoparticles [11]. To investigate the alterations in the bioactive profile of the nanoparticles induced after the manipulation of the surface charge, we selected Ag(28)@mSiO_2_ as a model, considering its lower biocidal effect.

Upon functionalization with APTES, we successfully obtained positively charged Ag(28)@mSiO_2_-NH_2_ nanoparticles, as previously detailed, featuring a ζ-potential of +36.6 mV. The impact of this functionalization on the bioactive profile of the nanoparticles is evident through the MIC values obtained for various strains, indicating a significant decrease in all cases. Notably, this effect is particularly pronounced against *Salmonella Choleraesuis*, where the MIC and MBC were measured at 4.5 µg/mL. In contrast, the larger and smaller negatively charged particles showed higher values of inhibition with an associated bactericidal effect, at a concentration of 18.2 µg/mL, resulting in a 4-fold reduction in the nanoparticle concentration necessary for an inhibitory and bactericidal antimicrobial efficacy. Additionally, a 2-fold reduction in the positively charged particles was observed against *B. cereus*.

Ag(28)@mSiO_2_-NH_2_ nanoparticles, present competitive concentrations with others reported in the literature for silver nanoparticles. For example, some studies point to the occurrence of MICs in the range of 100 to 25 μg/mL for *E. coli* [51,52] in other silver nanoparticles coated with a mesoporous shell, which are above those reported herein at a concentration of 18.2 μg/mL. However, to the best of our knowledge, no studies have been performed for mesoporous silica shell-silver core nanoparticles against *B. cereus* and *S. enterica*. As such, a comparative analysis can only be performed against simple silver nanoparticles. As an example, for *B. cereus* and *S. enterica*, some studies point to MIC values of 12.5 μg/mL [53] and 19.5 μg/mL [54]. Although the direct comparison between these values and those obtained for this study is not possible due to the presence of the mesoporous silica shell, it is possible to notice the benefits of the introduction of a silica shell in the herein reported nanoparticles, particularly in the case of *S. enterica*, as we obtained inhibitory concentrations fairly lower than those reported, even with a mesoporous shell. This is yet another indicative factor of the fact that the presence of a mesoporous shell can help in the stabilization of the nanoparticles, without hindering their antibacterial activity.

These findings highlight an enhanced inhibitory activity, especially against *Salmonella Choleraesuis*, with a clear improvement in effectiveness from positively charged particles. It is worth noting that bacteria typically possess a negative charge on their surface, often due to the presence of a lipopolysaccharide-rich (LPS) outer membrane in Gram-negative bacteria [55], or by the presence of negatively charged teichoic acids in the membrane of Gram-positive bacteria [56]. This negative charge in bacterial cells may allow for easier electrostatic-attractive interactions with the positively charged NPs and Ag(+) ions, leading to a disruption of the cell envelope and several cytosolic processes such as the denaturation of ribosomes and interference with DNA replication processes, amongst others, ultimately resulting in bacterial death [57,58].

Although the precise mechanism by which the particles herein reported induce an inhibitory activity is not yet known and requires further research [59], it is clear that a positive surface modification allows for an easier interaction between membranes and nanoparticles [60], potentiating their antibacterial effects.

So, as the positively charged Ag(28)@mSiO_2_-NH_2_ NPs were able to produce a very pronounced antibacterial effect against all tested strains, with a particular emphasis on *Salmonella* Choleraesuis with a MIC and MBC of only 4.5 µg/mL, the previously manufactured doped IROGRAN^®^ polymers were also tested for their antibacterial activity.

**Figure 7:**
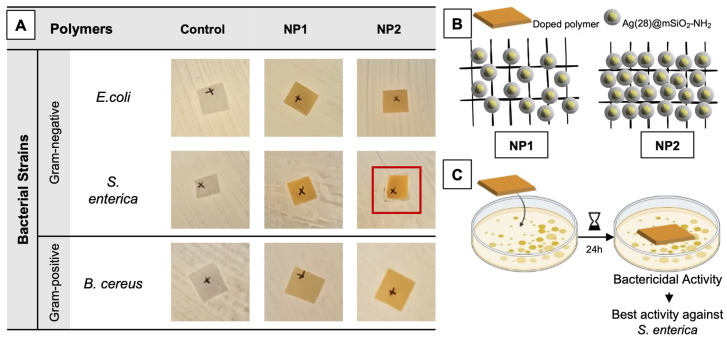
Assessment of antibacterial activity of Ag(28)@mSiO_2_-NH_2_-doped IROGRAN polymers (**A**). Polymers were doped with a lower (NP1) and higher (NP2) NP concentration, respectively. Bacterial strains selected were *Escherichia coli* (*E. coli*, ATCC^®^ 25922^TM^), *Salmonella enterica* subsp. *enterica* serovar Choleraesuis (ATCC^®^ 10708^TM^) and *Bacillus cereus* (ATCC^®^ 11778^TM^). Areas of all polymeric squares ca. 1 cm^2^. Red highlight indicates the best obtained results, through the occurrence of a slight inhibitory halo. Schematic overview of the different polymeric matrixes applied (**B**) and the assay conducted (**C**).

Three different samples were evaluated: non-doped control polymers and the polymers doped with two concentrations of the positively charged nanoparticles, denoted here as NP1 and NP2, respectively. As seen in Figure 7A, the control polymers were not able to produce any bactericidal activity, as bacterial growth was verified in the polymer–agar interface. As such, the antibacterial activity that the doped polymers produce is derived from the nanoparticles themselves and not from the polymeric matrix. Considering the doped polymers, although two different nanoparticle concentrations were tested (Figure 7B), there was not any significant difference between the obtained antibacterial results. Even with the lower nanoparticle concentration, a full bactericidal activity was observed, with no bacterial growth verified in the polymer–agar interface. However, an interesting result was obtained for *Salmonella* Choleraesuis. When in the presence of the polymer with the higher nanoparticle concentration, NP2, a slight inhibitory halo was observed, as highlighted in red in Figure 7A. This slight halo indicates that the polymers may have a stronger bactericidal activity against *Salmonella* Choleraesuis in comparison to the other tested strains. These results also corroborate the ones obtained for the liquid assays, in which the MIC/MBC obtained for *Salmonella* Choleraesuis was the lowest determined (MIC/MBC = 4.5 µg/mL). Moreover, this bactericidal activity was verified for both Gram-positive and Gram-negative bacteria, once again demonstrating the broad-spectrum in the antibacterial activity of these nanoparticles, with and without a solid support matrix.

The exact process through which antibacterial effects are generated remains uncertain. However, it is evident that embedding these particles into a polymer matrix in a stable and uniform way does result in the creation of solid and flexible surfaces with potent antibacterial activity.

Moreover, it appears that the incorporation of silver nanoparticles onto a solid-support matrix did not hinder its silver ion release capacity, as a clear bactericidal activity was observed. The synergy between the various mechanisms that silver ions produce in an intracellular bacterial environment result in a highly effective and stable polymer for bacterial control, with potential applicability in a variety of fields, from clinical to food-packaging.

## 4. Conclusions

A precise manufacturing method has been documented to produce significant quantities of well-defined mesoporous silica-coated silver and gold nanoparticles at high yields. The versatility of this coating technique has been confirmed on metal cores of different sizes or composition, resulting in a four-fold increase in nanoparticle production compared to our previous efforts. It is important to highlight that this scalability did not compromise the efficiency of the reaction, producing well-defined mesoporous silica-coated silver or gold nanoparticles in high yield. Subsequent derivatization with APTES successfully produced amine-terminated nanoparticles. Furthermore, their incorporation into thermoplastic polyurethane films via solution casting was successfully achieved, allowing the fabrication of films with variable nanoparticles concentrations. The exploration of the antibacterial properties of Ag@mSiO_2_ NPs, of two different sizes and with different surface functionalization, as well as Au@mSiO_2_ NPs, yielded interesting results. Our findings suggest that the mesoporous silica coating does not impede the antibacterial efficacy of silver nanoparticles, exhibiting a size-dependent effect. Surprisingly, we confirmed that by manipulating the surface charge of NPs through APTES functionalization, their antimicrobial efficacy was significantly improved. Specifically, the most powerful antibacterial and bactericidal effect was observed in *Salmonella* Choleraesuis strains for Ag(28)@mSiO_2_-NH_2_, with the lowest MIC/MBC recorded at 4.5 µg/mL. The remarkable antibacterial efficacy demonstrated by mesoporous silica-coated silver nanoparticles against Gram-negative bacteria strains, together with the discernible impact of core size and surface charge on bactericidal activity, underscores their potential to enhance the development of materials with antimicrobial properties. Furthermore, the seamless integration of these nanoparticles into polymeric materials further accentuates their versatility and provides valuable information to refine the design of engineered surfaces with antimicrobial attributes, with potential applications in various sectors including healthcare and the food industry.

## Data Availability

Data are contained within the article and Supplementary Materials.

## Supplementary Materials

The following supporting information can be downloaded at: https://www.mdpi.com/article/10.3390/nano14050462/s1. Figure S1: (A) Normalized extinction spectra of Ag(28) NPs, Ag(51) NPs and Au NPs, size distribution of (B) Ag(28) NPs, (C) Ag(51) NPs and (D) Au NPs. Figure S2: Extinction spectra of (A) Ag(51)@mSiO_2_ and (B) Au@mSiO_2_ during different purification cycles in MEOH and water demonstrating the blue shift of the LSPR band. Figure S3: (A) ζ-potential of Ag(28)@mSiO_2_, (B) Ag(51)@mSiO_2_ and (C) Au@mSiO_2_ during different purification cycles in MeOH and water. Figure S4. HRTEM images and SAED pattern of AgNPs with 28 nm (A,B) and 51 nm (C,D) showing similar polycrystalline structure. Figure S5. Survey spectra of Ag(28)@mSiO_2_ NPs (A) and Ag(51)@mSiO_2_ NPs (B). Figure S6. Enlarged XPS spectra in the Ag 3d region, deconvolution of the Ag 3d and spectra and enlarged spectra in the O 1s region for Ag(28)@mSiO_2_ NPs (A,B,C, respectively) and Ag(51)@mSiO_2_ NPs (D,E,F, respectively).
