# Supplementary material for: Tailoring Mesoporous Silica-Coated Silver Nanoparticles and Polyurethane-Doped Films for Enhanced Antimicrobial Applications
Source: Nanomaterials (Basel). 2024 Mar 2;14(5):462. doi: 10.3390/nano14050462 (PMC10934637; doi:10.3390/nano14050462)
Supplement: Supplementary file 1 [file nanomaterials-14-00462-s001.zip › nanomaterials-2869591-supplementary.pdf]

Tailoring Mesoporous Silica-Coated Silver Nanoparticles and Polyurethane-Doped Films for Enhanced Antimicrobial Applications

Silvia Nuti [1,2], Adrián Fernández-Lodeiro [1,2], Joana Galhano [1,2], Elisabete Oliveira [1,2,*], Maria Paula Duarte [3], José Luis Capelo-Martínez [1,2], Carlos Lodeiro [1,2] and Javier Fernández-Lodeiro [1,2,*]

[1] BIOSCOPE Research Group, LAQV-REQUIMTE, Chemistry Department, NOVA School of Science and Technology (FCT NOVA), Universidade NOVA de Lisboa, 2829-516 Caparica, Portugal; s.nuti@campus.fct.unl.pt (S.N.); a.lodeiro@campus.fct.unl.pt (A.F.-L.); j.galhano@campus.fct.unl.pt (J.G.); jlcm@fct.unl.pt (J.L.C.-M.); cle@fct.unl.pt (C.L.)
[2] PROTEOMASS Scientific Society, Praceta Jeronimo Dias, Num. 12, 2A, Sto Antonio de Caparica, 2825-466 Costa de Caparica, Portugal
[3] MEtRICs, Chemistry Department, NOVA School of Science and Technology (FCT NOVA), Universidade NOVA de Lisboa, 2829-516 Caparica, Portugal; mpcd@fct.unl.pt
* Correspondence: ej.oliveira@fct.unl.pt (E.O.); j.lodeiro@fct.unl.pt (J.F.-L.)

Supporting information

1. Additional Figures

Figure S1: (A) Normalized extinction spectra of Ag(28) NPs, Ag (51) NPs and Au NPs, size distribution of (B) Ag(28) NPs, (C) Ag(51) NPs and (D) Au NPs……………………………………………………………………………2

Figure S2: Extinction spectra of (A) Ag(51)@mSiO_2 and (B) Au@mSiO_2 during different purification cycles in MEOH and water demonstrating the blue shift of the LSPR band……………………………………3

Figure S3: (A) ζ-potential of Ag(28)@mSiO_2, (B) Ag(51)@mSiO_2 and (C) Au@mSiO_2 during different purification cycles in MeOH and water……………………………………………………………………………………4

Figure S4. HRTEM images and SAED pattern of AgNPs with 28nm (A, B) and 51 nm (C, D) showing similar polycrystalline structure……………………………………………………………………………………………….4

Figure S5. Survey spectra of Ag(28)@mSiO_2 NPs (A) and Ag(51)@mSiO_2 NPs (B)……………………………….4

Figure S6. Enlarged XPS spectra in the Ag 3d region, deconvolution of the Ag 3d spectra and enlarged spectra in the O 1s region for Ag(28)@mSiO_2 NPs (A, B, C respectively) and Ag(51)@mSiO_2 NPs (D, E, F respectively)……….5

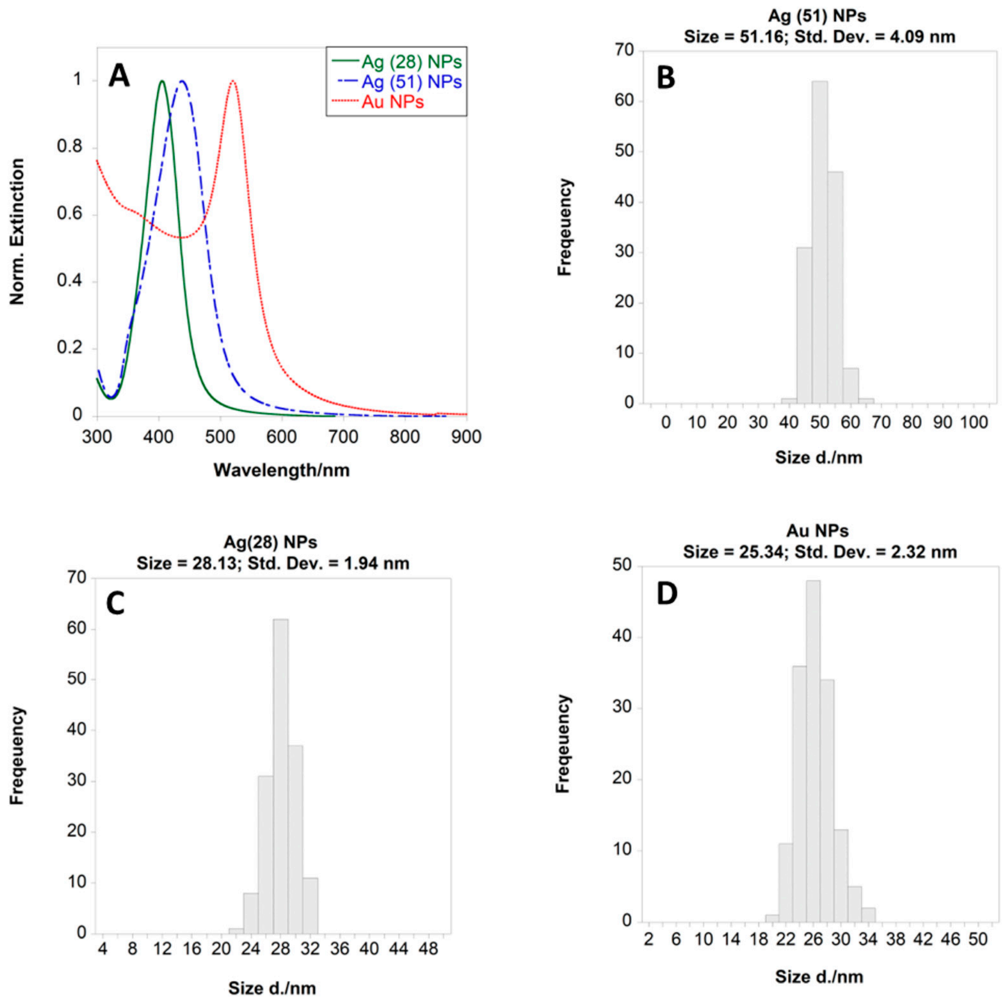

Figure S1: (A) Normalized extinction spectra of Ag(28) NPs, Ag(51) NPs and Au NPs, size distribution of (B) Ag(28) NPs, (C) Ag(51) NPs and (D) Au NPs.

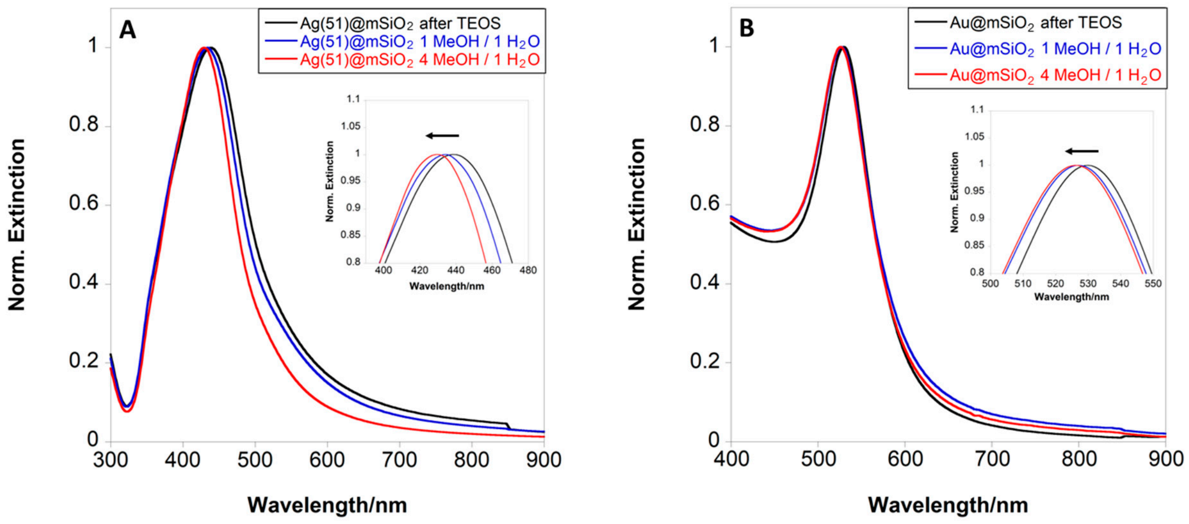

Figure S2: Extinction spectra of (A) Ag(51)@mSiO_2 and (B) Au@mSiO_2 during different purification cycles in MEOH and water demonstrating the blue shift of the LSPR band.

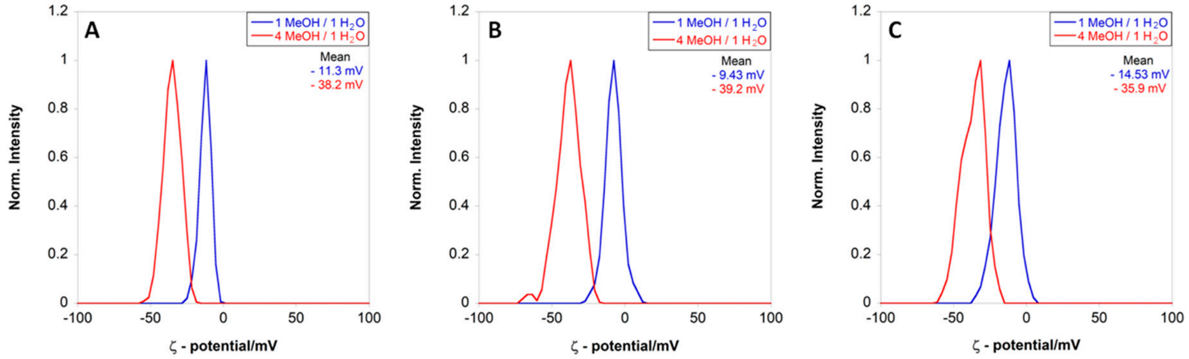

Figure S3: (A) ζ-potential of Ag(28)@mSiO_2, (B) Ag(51)@mSiO_2 and (C) Au@mSiO_2 during different purification cycles in MeOH and water.

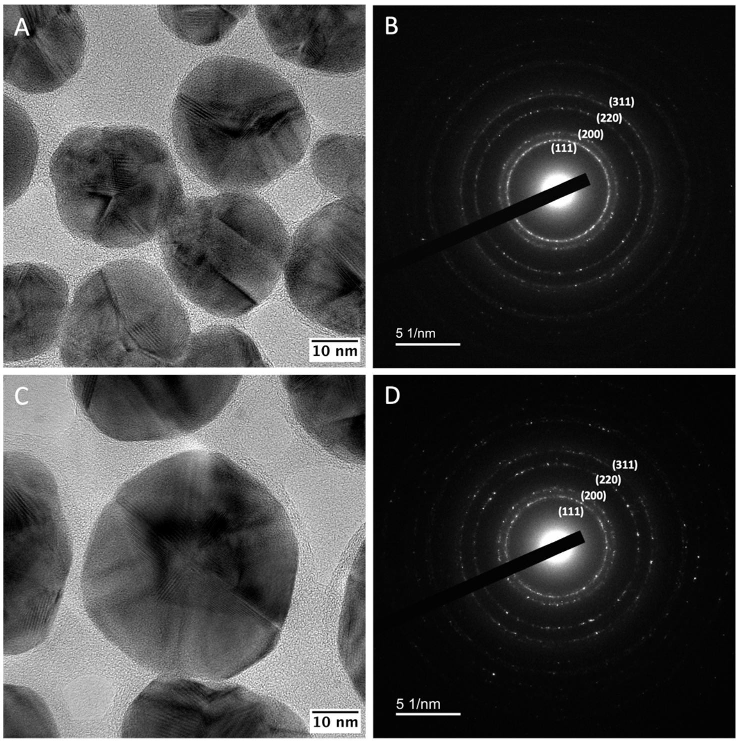

Figure S4. HRTEM images and SAED pattern of AgNPs with 28nm (A, B) and 51 nm (C, D) showing similar polycrystalline structure.

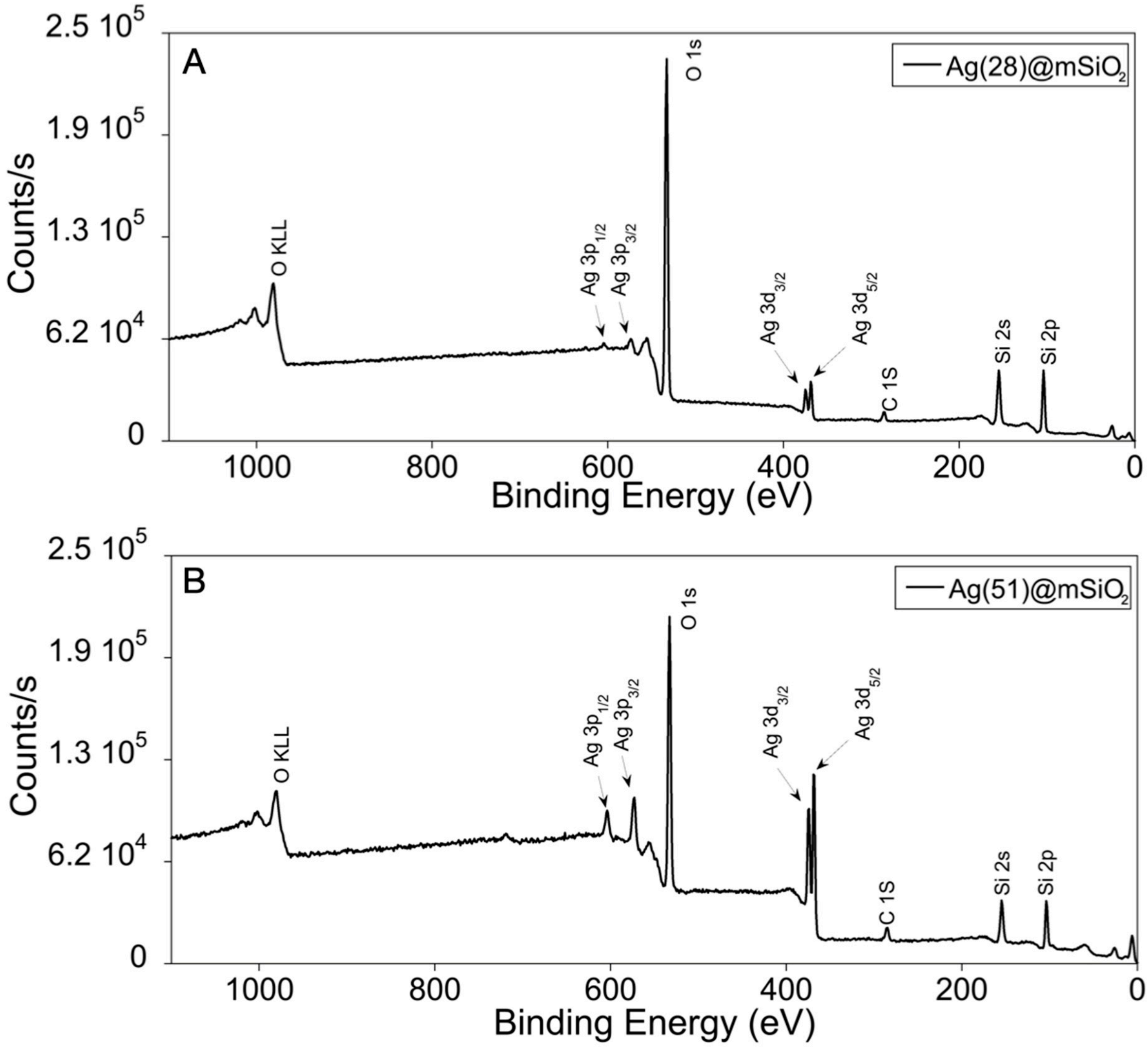

Figure S5. Survey spectra of Ag(28)@mSiO_2 NPs (A) and Ag(51)@mSiO_2 NPs (B)

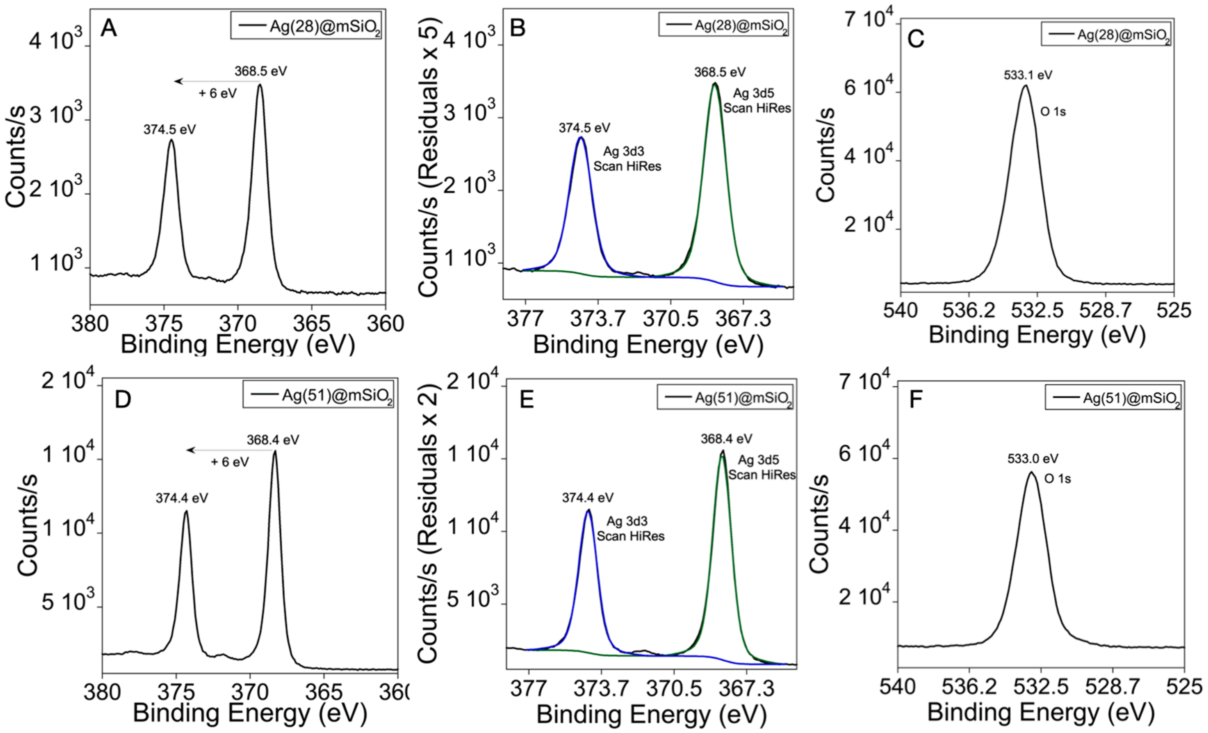

Figure S6. Enlarged XPS spectra in the Ag 3d region, deconvolution of the Ag 3d spectra and enlarged spectra in the O 1s region for Ag(28)@mSiO_2 NPs (A, B, C respectively) and Ag(51)@mSiO_2 NPs (D, E, F respectively).
